# Dietary magnesium intake modified association between visceral adiposity index and chronic kidney disease: a cross-sectional study

**DOI:** 10.29219/fnr.v70.14223

**Published:** 2026-09-24

**Authors:** Xue Wang, Xiuxia Han, Rongzhen Kong

**Affiliations:** Department of Nephrology, Qilu Hospital of Shandong University, Dezhou Hospital, Dezhou, China

**Keywords:** chronic kidney disease, visceral adiposity index, dietary magnesium intake, logistic regression, curve fitting

## Abstract

**Background:**

Chronic kidney disease (CKD) is considered as one of the major global public health concerns. The visceral adiposity index (VAI), a marker of visceral fat dysfunction, has been associated with CKD in previous reports; however, evidence from U.S. populations remains limited. In addition, the potential modifying role of dietary magnesium intake in this association is not well understood. This study aimed to investigate the relationship between VAI and CKD among U.S. adults and to explore whether dietary magnesium intake modifies this association.

**Methods:**

A total of 2,616 participants from the National Health and Nutrition Examination Survey (NHANES) between 2003 and 2018 were included in this retrospective study. The association between VAI and CKD was evaluated using curve-fitting analyses, multivariable logistic regression models, and subgroup analyses stratified by dietary magnesium intake.

**Results:**

A significant positive relation between VAI and CKD was observed only among individuals with low dietary magnesium intake. In this group, each one-unit increase in VAI was associated with higher odds of CKD (odds ratio [OR] = 1.13, 95% confidence interval [CI]: 1.07–1.18; *P* < 0.001) after adjustment for potential confounders. In contrast, no significant relation was observed among individuals with high dietary magnesium intake (OR = 1.03, 95% CI: 0.96–1.10; *P* = 0.252).

**Conclusions:**

Higher VAI is significantly associated with an increased risk of CKD among U.S. adults with low dietary magnesium intake. Adequate magnesium consumption may attenuate the adverse association between visceral adiposity and kidney health.

## Popular scientific summary

Higher levels of hidden belly fat were linked to a greater risk of chronic kidney disease – but only in people who consumed low amounts of magnesium. In those with higher magnesium intake, this risk was not observed. This suggests that magnesium-rich foods may help protect kidney health, especially in individuals with excess abdominal fat.

Advances and improvements in medical technology and knowledge over recent decades have shifted the global disease burden from infectious diseases toward non-communicable diseases (NCDs) (1). Chronic kidney disease (CKD) has become a major public health problem in both developed and developing countries and is associated with the large increase in the prevalence of diabetes mellitus, hypertension and obesity (2–6). It is reported that CKD is responsible for 2.53% of the global disease deaths in 2019, while the proportions of years of life lost and disability-adjusted life years were estimated at 1.09 and 1.64%, respectively (7). In the U.S., around 37 million adults have CKD (8) and are at higher risk of cardiovascular events, kidney failure and death (9).

It is estimated that the adverse effects of CKD were much higher in some developing countries. For example, CKD accounted for 10.15 and 11.89% of all deaths in El Salvador and Nicaragua, respectively. The percentage of life lost were 6.47 and 7.07%, while the reported lifetime with disability was 2.24 and 2.28%, respectively (10). Therefore, it seems that identifying modifiable risk factors for CKD is of considerable public health importance.

The visceral adiposity index (VAI) is an empirical mathematical model widely used to assess visceral fat distribution and dysfunction (11). VAI is calculated using anthropometric measurements, including body mass index (BMI) and waist circumference, together with laboratory parameters such as triglycerides (TG) and high-density lipoprotein cholesterol (HDL-C) (12). Previous studies have reported associations between elevated VAI and several metabolic and endocrine disorders, including acromegaly (13), polycystic ovary syndrome (14), type 2 diabetes mellitus (15, 16), and prolactinoma (17). Therefore, VAI has been considered an important and useful indicator for evaluating metabolic health and predicting disease risk.

Although CKD and visceral fat are both important health problems and can influence each other, evidence regarding the relation or association between VAI and CKD remains inconsistent. Seong et al. (18) evaluated the relationship between VAI and CKD in a population of 4,947 Korean adults, and found a positive correlation after adjustment for potential confounding factors in men. In a similar retrospective study by Chen et al. (19), performed on 23,570 Taiwanese individuals, the association of VAI with CKD was found to be significant in men (odds ratio [OR] = 1.62, 95% confidence interval [CI]: 1.13–2.32) but not in women (OR = 1.28, 95% CI: 0.66–2.47; *P* = 0.469). Furthermore, in a study on the longitudinal data set of the China Health and Retirement Longitudinal Study (20), it was reported that an increased risk of CKD was seen with higher VAI scores in males but not in females. These studies offer important evidence but the majority of the studies have been performed among Asian populations and may not be completely applicable to other ethnic groups. Furthermore, large-scale nationally representative studies evaluating the association between VAI and CKD in U.S. adults remain scarce. Therefore, additional research is needed to better determine the relationship between VAI and CKD in U.S. American adults. Moreover, whether dietary magnesium intake modifies this association has not been investigated previously in the U.S. population on a large scale.

Magnesium is among the most prevalent minerals in the human body (21), and is involved in many physiologic functions (22–24). It is a cofactor of enzymes that are crucial in the synthesis of RNA and DNA, cellular repair, and antioxidant defense systems (25, 26). Furthermore, magnesium plays an essential role in vitamin D metabolism and activation, and vitamin D has been reported to exert protective effects against CKD in several studies (27).

Previous studies have indicated that the consumption of diets rich in magnesium can affect the risk of insulin resistance, diabetes, and metabolic syndrome (28–30). The different metabolic roles played by magnesium could be responsible for these effects. In addition, magnesium homeostasis is closely associated with kidney function. Eventually, CKD causes changes in magnesium handling and excretion that can lead to changes in magnesium serum levels, which can affect clinical outcomes (31, 32). Although these observations were made, it remains elusive as to whether dietary magnesium intake affects the association between visceral adiposity and CKD.

The purpose of this study was thus to examine the relationship between VAI and CKD in a nationally representative sample of adults in the U.S. population and to determine whether the dietary magnesium intake modifies the relationship between VAI and CKD. We hypothesized that adequate dietary magnesium intake may attenuate the positive association between visceral adiposity and CKD.

## Methods

### Data sources

The data were collected from the National Center for Health Statistics (NCHS). This dataset was provided through the National Health and Nutrition Examination Survey (NHANES), which is a nationally representative survey aimed at measuring the health and nutritional status of the U.S. population (33, 34). The sampling design of NHANES was multistage, stratified probability sampling technique to provide nationally representative estimates. For the present analysis, data from eight consecutive survey cycles (2003–2004 to 2017–2018) were pooled together, including the demographic, dietary, laboratory, examination, and questionnaire data. Detailed survey protocols, laboratory procedures, and ethics information are publicly available on the NHANES website (https://www.cdc.gov/nchs/nhanes/). Written informed consent was obtained from all participants and the survey protocol was approved by the NCHS Research Ethics Review Board.

### Study design and participants

The participants were included in the study with the following criteria: The age of ≥ 20 years; successful completed Mobile Examination Center (MEC) exam; available data for VAI calculation; complete information on CKD status; and available dietary magnesium intake data. Participants with missing data on VAI components, CKD status, dietary magnesium intake, or other key study variables were excluded from the samples. Figure 1 shows the steps of participant’s selection procedure.

**Fig. 1:**
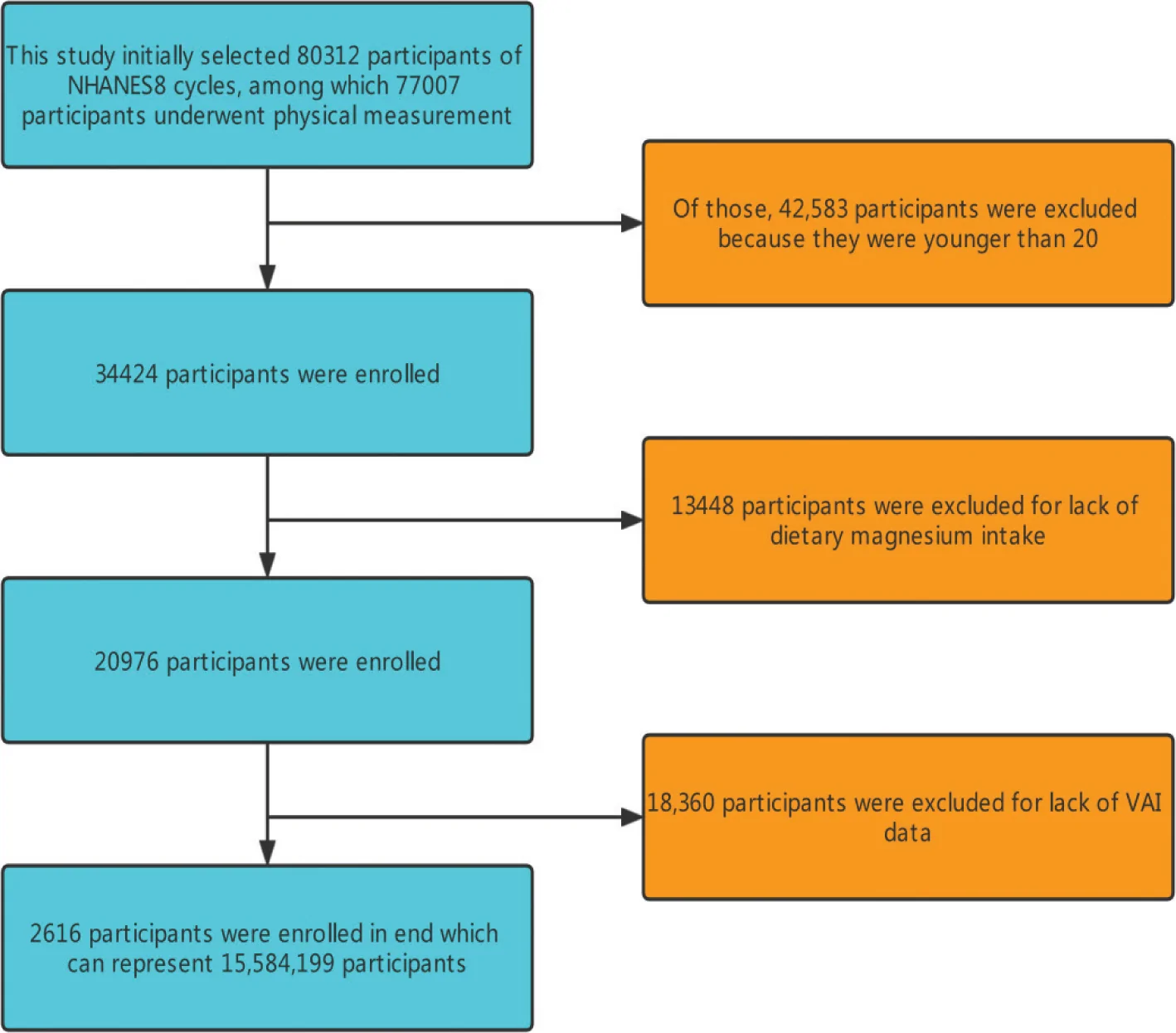
Flowchart of participant selection.

### Data collection

Trained researchers collected and recorded all data according to standardized NHANES protocols. The evaluated variables were VAI, CKD status, demographic variables, lifestyle variables, dietary variables and laboratory variables. The considered covariates were age, sex, race/ethnicity, education level, smoking status, alcohol intake, physical activity, energy intake, hypertension, diabetes, alanine aminotransferase (ALT), aspartate aminotransferase (AST), blood urea nitrogen (BUN), lactate dehydrogenase (LDH), and serum albumin (ALB).

#### Visceral adiposity index

VAI, which is a sex-specific visceral fat distribution/dysfunction indicator, is an index considered the combination of anthropometric and metabolic parameters. The following equations were used to calculate VAI:

For men:

$$VAI=\left(\frac{WC}{39.68+1.88\times BMI}\right)\times \left(\frac{TG}{1.03}\right)\times \left(\frac{1.31}{HDL}\right)$$

For women:

$$VAI=\left(\frac{WC}{39.58+1.89\times BMI}\right)\times \left(\frac{TG}{0.81}\right)\times \left(\frac{1.52}{HDL}\right)$$

where WC represents waist circumference (cm), BMI is body mass index (kg/m^2^), TG is triglycerides level (mmol/L), and HDL-C represents high-density lipoprotein cholesterol (mmol/L). Trained researchers performed the measurements of body weight, height, and waist circumference, following standard NHANES procedures. BMI was computed as weight (kg)/height squared (m^2^). Laboratory measurement of TG and HDL-C were conducted following NHANES laboratory procedures. More information can be found in the NHANES Laboratory Procedures Manuals.

#### Measurement of CKD

The criteria for the diagnosis of CKD were based on the kidney disease: Improving Global Outcomes (KDIGO) criteria (35). Those who were identified as having CKD must have met at least one of the following criteria (36):

The estimated glomerular filtration rate (eGFR) < 60 mL/min/1.73 m^2^ for more than 3 months; or urine albumin to creatinine ratio (UACR) ≥ 30 mg/g in two of three spot urine specimen.

The eGFR was calculated using the Modification of Diet in Renal Disease (MDRD) equation:

$$\text{eGFR}=186\times {\left(\text{Scr}\right)}^{-1.154}\times {\left(\text{Age}\right)}^{-0.203}\times (0.742\text{ if female}),$$

where Scr is serum creatinine concentration (mg/dL). Urinary albumin and creatinine concentrations were measured using standardized NHANES laboratory procedures, and UACR was calculated as urinary albumin (mg/L) divided by urinary creatinine (g/L).

#### Assessment of dietary magnesium intake

Dietary magnesium intake was assessed using the first 24-h dietary recall interview conducted by trained NHANES interviewers as part of the What We Eat in America (WWEIA) survey, using the automated multiple-pass method. Magnesium intake was estimated from all foods and beverages consumed during the previous 24 h.

The cutoff value of 270 mg/day corresponded to the weighted median dietary magnesium intake in the study population, calculated using the NHANES sample weights. Participants were categorized into low (< 270 mg/day) and high (≥ 270 mg/day) magnesium intake groups. This approach has been widely used in previous NHANES-based nutritional epidemiology studies to evaluate potential interaction effects between nutrient intake and other modifiers (28–30).

#### Measurement of covariates

Race/ethnicity was defined as Mexican American, Non-Hispanic White, Non-Hispanic Black, Non-Hispanic Asian, other Hispanic, and other race. Education attainment was categorized as less than high school, high school or higher education.

Smoking status was classified as never smokers, former smokers, and current smokers. Alcohol consumption was defined as never drinker, former drinker, mild drinker, moderate drinker, and heavy drinker based on NHANES definitions.

Hypertension and diabetes status were classified based on responses to questions on questionnaires, examination, and laboratory measurements following NHANES protocols.

Confounding variables were chosen from previous literature (11, 18–20), based on biological plausibility and their known relationships with VAI and CKD. Not all medication-use variables (e.g. antihypertensive agents, lipid-lowering drugs) and socioeconomic indicators were consistently available across all survey cycles.

### Statistical methods

All analyses incorporated NHANES sampling weights, strata (SDMVSTRA), and primary sampling units (SDMVPSU) to account for the complex multistage sampling design and to generate nationally representative estimates.

Data were analyzed statistically using the R software (version 4.2.4; R Foundation for Statistical Computing, Vienna, Austria). Values of continuous variables with normal distributions were reported as weighted means ± standard deviations and those of skewed variables as weighted medians and interquartile ranges. Weighted frequencies and percentages were used for categorical variables.

Multiple imputation by chained equations (MICE) was used to deal with missing covariate data. Five imputed datasets were created and combined using the Rubin’s rules. Variables that were used in the imputation model comprised all exposure, outcome, and covariate variables in the main analyses. The results from sensitivity analyses of imputed and complete-case data sets showed no significant differences. The factors associated with CKD were initially investigated using univariable logistic regression analyses. Multivariable logistic regression models were then created to assess the relationship between VAI and CKD.

Model 1: Unadjusted. Model 2: Adjusted for age, sex, race/ethnicity, and education. Model 3: Further adjusted for physical activity, smoking, alcohol intake, energy intake, hypertension and diabetes. Model 4: Additionally adjusted for ALT, AST, BUN, LDH, and ALB.

The multicollinearity of covariates was examined by variance inflation factors (VIFs) and no significant multicollinearity was found. Four knots at the 5th, 35th, 65th, and 95th percentile of the VAI distribution were used to test for possible non-linear relationship between VAI and CKD using restricted cubic spline (RCS) analyses. Likelihood ratio tests were used for the assessment of non-linearity. Possible effect modification was explored through subgroup analysis by the dietary magnesium intake. Sensitivity analysis was performed by dividing VAI into quartiles and running linear trend tests by quartiles. All statistical tests were performed two-sided and *P* < 0.05 was regarded as statistically significant.

## Results

### Basic information of participants

A total of 2,616 participants were included in the final analysis, representing approximately 15.6 million U.S. adults after applying the NHANES sampling weights. Females and males accounted for 52.75 and 47.25% of the weighted population, respectively. The baseline characteristics of the study population are presented in Table 1.

**Table 1:** Basic characteristics of participants (weighted)

Variable	Total	Non-CKD	CKD	*P*
Age (years)	55.17 ± 0.43	52.92 ± 0.49	67.72 ± 0.74	< 0.0001
BMI (kg/m^2^)	28.63 ± 0.19	28.39 ± 0.20	29.96 ± 0.48	0.002
Waist circumference (cm)	99.88 ± 0.48	99.09 ± 0.49	104.29 ± 1.15	< 0.0001
Magnesium intake (mg/day)	127.27 ± 6.59	128.78 ± 7.37	118.88 ± 9.45	< 0.001
Energy intake (kcal/day)	2140.79 ± 25.70	2198.24 ± 29.02	1819.84 ± 44.00	< 0.0001
ALT (U/L)	22.00 (17.00–29.00)	22.00 (17.00–29.00)	20.00 (16.00–27.00)	< 0.001
AST (U/L)	24.00 (21.00–28.00)	24.00 (21.00, 28.00)	24.00 (21.00–29.00)	0.35
BUN (mg/dL)	5.00 (3.93–6.07)	5.00 (3.93–5.71)	6.07 (5.00–8.21)	< 0.0001
LDH (U/L)	131.00 (115.00–149.00)	130.00 (114.00–146.00)	141.00 (125.00–165.00)	< 0.0001
ALB (g/L)	43.00 (41.00–45.00)	43.00 (41.00–45.00)	42.00 (40.00–44.00)	< 0.0001
VAI	1.87 ± 0.05	1.78 ± 0.04	2.36 ± 0.15	< 0.0001
Sex				0.11
Female	52.75 (48.07–57.44)	83.77 (81.73–85.81)	16.23(14.19, 18.27)	
Male	47.25 (42.80–51.69)	85.98 (83.86–88.11)	14.02(11.89, 16.14)	
Race/Ethnicity				0.46
Mexican American	4.09 (3.26–4.91)	86.16 (81.26–91.06)	13.84 (8.94–18.74)	
Non-Hispanic Black	5.71 (4.76–6.66)	81.50 (77.72–85.28)	18.50 (14.72–22.28)	
Non-Hispanic White	80.82 (72.82–88.82)	85.18 (83.40–86.95)	14.82 (13.05–16.60)	
Other Hispanic	3.46 (2.68–4.25)	82.46 (76.11–88.81)	17.54 (11.19–23.89)	
Other Race – Including Multiracial	5.92 (4.64–7.21)	83.54 (78.00–89.08)	16.46 (10.92–22.00)	
Activity				< 0.0001
No	39.62 (35.21–44.04)	78.59 (75.45–81.73)	21.41 (18.27–24.55)	
Moderate	33.94 (29.88–38.00)	86.44 (83.87–89.00)	13.56 (11.00–16.13)	
Vigorous	26.43 (23.08–29.79)	92.06 (89.40–94.73)	7.94 (5.27–10.60)	
Smoke				0.35
Former	33.32 (29.08–37.56)	82.79 (79.24–86.34)	17.21 (13.66–20.76)	
Never	54.18 (49.46–58.91)	85.65 (83.47–87.82)	14.35 (12.18–16.53)	
Now	12.49 (10.64–14.35)	86.63 (81.24–92.02)	13.37 (7.98–18.76)	
Alcohol consumption				< 0.0001
Former	12.60 (10.33–14.87)	78.21 (72.32–84.10)	21.79 (15.90–27.68)	
Heavy	13.34 (11.65–15.03)	92.64 (89.32–95.96)	7.36 (4.04–10.68)	
Mild	42.11 (37.31–46.91)	85.75 (83.11–88.39)	14.25 (11.61–16.89)	
Moderate	15.28 (12.66, 17.91)	89.17 (85.13, 93.20)	10.83 (6.80, 14.87)	
Never	9.49 (7.29, 11.69)	75.66 (70.34, 80.98)	24.34 (19.02, 29.66)	
Hypertension				< 0.0001
No	54.35 (48.90–59.80)	93.95 (92.58–95.32)	6.05 (4.68–7.42)	
Yes	45.65 (41.51–49.79)	73.94 (71.08–76.79)	26.06 (23.21–28.92)	
Diabetes				< 0.0001
No	79.61 (72.96–86.27)	88.81 (87.33–90.28)	11.19 (9.72–12.67)	
yes	19.31(16.84–21.79)	67.60 (62.77–72.43)	32.40 (27.57–37.23)	
Mg group				< 0.001
Low	49.18 (44.11–54.26)	83.73 (81.22–86.24)	16.27 (13.76–18.78)	
High	50.82 (46.32–55.32)	85.87 (83.59–88.15)	14.13 (11.85–16.41)	

There were no significant differences between race/ethnicity (*P* = 0.46), smoking status (*P* = 0.35), or AST levels (*P* = 0.35). These differences, however, were significant between alcohol consumption categories (all *P* < 0.001), hypertension status, diabetes status, level of physical activity and dietary level of magnesium (all *P* < 0.001).

Compared with participants without CKD, those with CKD had significantly higher BMI (29.96 vs. 28.39 kg/m^2^), waist circumference (104.29 vs. 99.09 cm), BUN (6.07 vs. 5.00 mg/dL), LDH (141.00 vs. 130.00 U/L), and VAI levels (2.36 vs. 1.78) (all *P* < 0.05). In contrast, dietary magnesium intake (118.88 vs. 128.78 mg/day), energy intake (1819.84 vs. 2198.24 kcal/day), ALT (20.00 vs. 22.00 U/L), and serum albumin levels (42.00 vs. 43.00 g/L) were significantly lower among participants with CKD (all *P* < 0.05).

### Violin plot analysis of differences in VAI levels

Figure 2 illustrates the distribution of VAI according to CKD status and dietary magnesium intake. Participants with high dietary magnesium intake exhibited lower VAI values than those with low magnesium intake in both the CKD and non-CKD groups (both *P* < 0.001). Furthermore, VAI levels were significantly higher among participants with CKD than among those without CKD regardless of magnesium intake category (all *P* < 0.001).

**Fig. 2:**
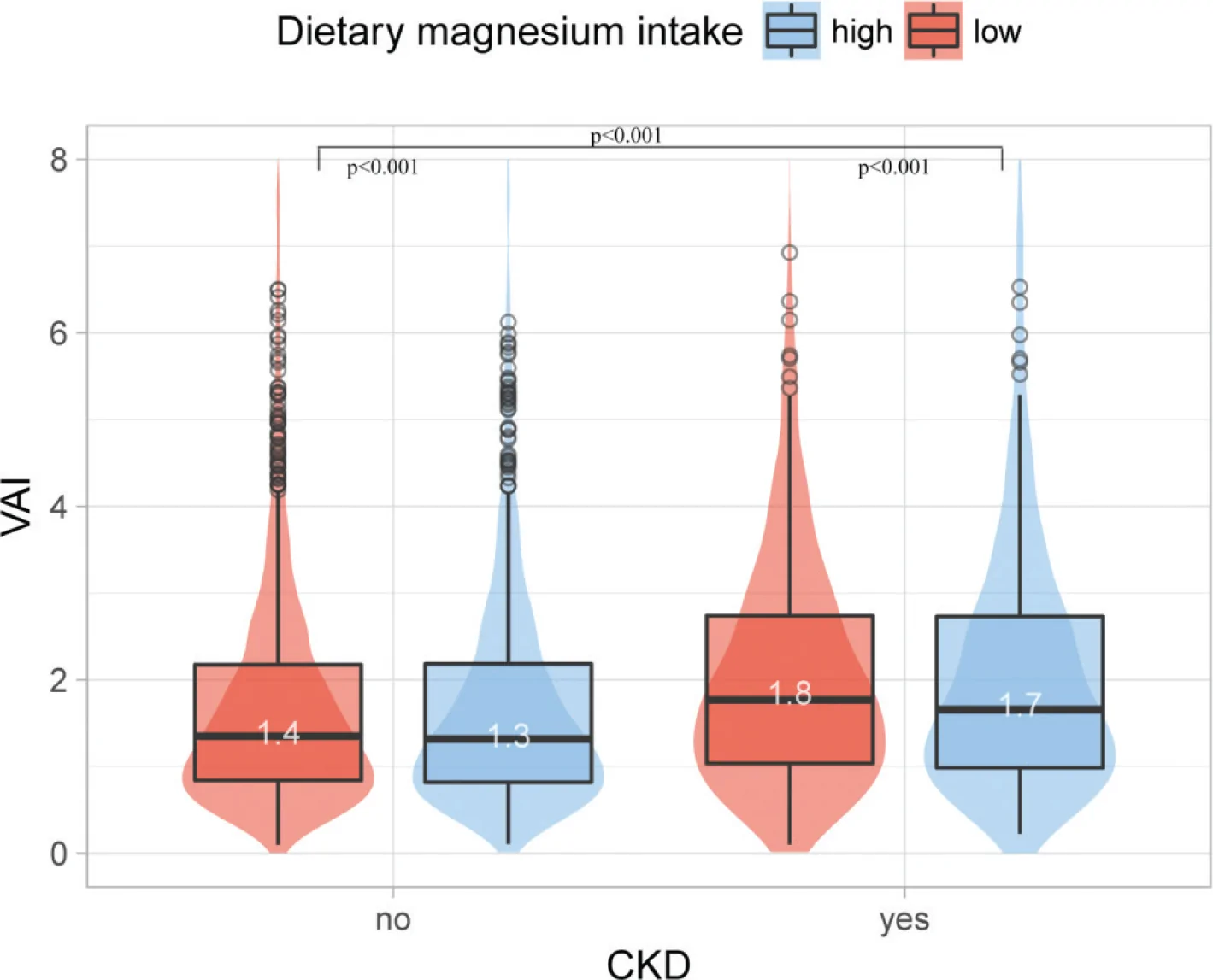
Distribution of visceral adiposity index (VAI) according to chronic kidney disease (CKD) status and dietary magnesium intake categories. Violin plots showing the distribution of VAI among participants with and without CKD, stratified by dietary magnesium intake (low vs. high). Participants with CKD exhibited higher VAI levels than those without CKD. Within both CKD and non-CKD groups, participants with higher dietary magnesium intake tended to have lower VAI levels. The width of each violin represents the density of observations.

### Univariate logistic regression analysis

Univariable logistic regression analyses were performed to determine the factors associated with CKD. CKD risk was positively related to age, BMI, waist circumference, BUN, LDH, hypertension and diabetes (all *P* < 0.05). In contrast, dietary magnesium intake, energy intake, physical activity, alcohol intake and serum albumin levels were negatively associated with CKD (all *P* < 0.05). There were no significant relationships noted for sex, race/ethnicity, smoking status, or AST (all *P* > 0.05) (Table 2).

**Table 2:** Univariate regression analysis and CKD-related variables (weighted)

Variable	OR (95% CI)	*P*
Age (years)	1.08 (1.07–1.10)	< 0.0001
BMI (kg/m^2^)	1.04 (1.02–1.06)	< 0.001
Waist circumference (cm)	1.02 (1.01–1.03)	< 0.0001
Magnesium (mg/day)	0.92 (0.90–0.94)	< 0.0001
Energy intake (kcal/day)	0.91(0.90–0.92)	< 0.0001
Sex		
Male	1	
Female	1.19 (0.96–1.47)	0.11
Race/Ethnicity		
Non-Hispanic White	1.08 (0.71–1.65)	0.71
Non-Hispanic Black	1.41(0.92–2.16)	0.11
Other Hispanic	1.32 (0.70–2.52)	0.39
Other Race – Including multiracial	1.23 (0.67–2.23)	0.51
Activity		
No	1	
Moderate	0.58 (0.43–0.77)	< 0.001
Vigorous	0.32 (0.20–0.49)	< 0.0001
Smoke		
Never	1	
Former	1.24 (0.90–1.71)	0.19
Now	0.92 (0.54–1.56)	0.76
Alcohol consumption		
Heavy	1	
Mild	2.09 (1.19–3.66)	0.01
Former	3.51 (1.96–6.27)	< 0.0001
Never	4.05 (2.16–7.59)	< 0.0001
Moderate	1.53 (0.79–2.98)	0.21
Hypertension (yes)		
No	1	
Yes	5.48 (4.23–7.10)	< 0.0001
DM (yes)		
No	1	
Yes	3.80 (2.96–4.88)	< 0.0001
ALT (U/L)	0.99 (0.98–1.00)	0.21
AST (U/L)	1.00 (1.00–1.01)	0.19
BUN (mg/dL)	1.53 (1.43–1.64)	< 0.0001
LDH (U/L)	1.01 (1.01–1.02)	< 0.0001
ALB (g/L)	0.91 (0.88–0.94)	< 0.0001

### Multivariate logistic regression analysis

In the unadjusted model (Model 1), VAI was positively associated with CKD (OR = 1.10, 95% CI: 1.04–1.17). Stratified analyses showed that this association was present only among participants with low dietary magnesium intake (OR = 1.16, 95% CI: 1.10–1.22), whereas no significant association was observed among those with high magnesium intake (OR = 1.05, 95% CI: 0.97–1.13).

The results remained consistent after adjustment for demographic, lifestyle, and biochemical covariates. In the fully adjusted model (Model 4), each one-unit increase in VAI was associated with 13% higher odds of CKD among participants with low dietary magnesium intake (OR = 1.13, 95% CI: 1.07–1.18; *P* < 0.001), whereas no significant association was observed among those with high dietary magnesium intake (OR = 1.03, 95% CI: 0.96–1.10; *P* = 0.252). A significant interaction between VAI and dietary magnesium intake was detected (*P* for interaction < 0.001) (Table 3).

**Table 3:** Multivariate logistic regression analysis of the association between VAI and CKD under different dietary magnesium intake levels (weighted)

Variable	Model 1			Model 2			Model 3			Model 4		
	OR (95% CI)	*P*	*P* for interaction	OR (95% CI)	*P*	*P* for interaction	OR (95% CI)	*P*	*P* for interaction	OR (95% CI)	*P*	*P* for interaction
VAI	1.10 (1.04–1.17)	< 0.001		1.10 (1.04–1.17)	< 0.001		1.08 (1.01–1.15)	< 0.001		1.08 (1.01–1.16)	< 0.001	
Mg intake group			< 0.001			< 0.001			< 0.001			< 0.001
Low	1.16 (1.10–1.22)	< 0.001		1.16 (1.10–1.22)	< 0.001		1.13 (1.07–1.19)	< 0.001		1.13 (1.07–1.18)	< 0.001	
High	1.05 (0.97–1.13)	0.139		1.05 (0.97–1.13)	0.142		1.03 (0.96–1.10)	0.252		1.03 (0.96–1.10)	0.252	

Model 1: Non – adjusted.

Model 2: Adjusted with age, sex, race/ethnicity, education.

Model 3: Adjusted with age, sex, race/ethnicity, education, activity, smoke, alcohol, energy, hypertension, diabetes.

Model 4: Adjusted with age, sex, race/ethnicity, education, activity, smoke, alcohol, energy, hypertension, diabetes, ALT, AST, BUN, LDH, ALB.

### RCS analysis

RCS analyses using the fully adjusted model (Model 4) were carried out to examine the dose-response association of VAI with CKD.

Overall, there was a positive linear association between VAI and CKD, without any evidence of non-linearity (*P* for non-linearity = 0.879) (Fig. 3). The risk of CKD increased progressively with increasing VAI levels.

**Fig. 3:**
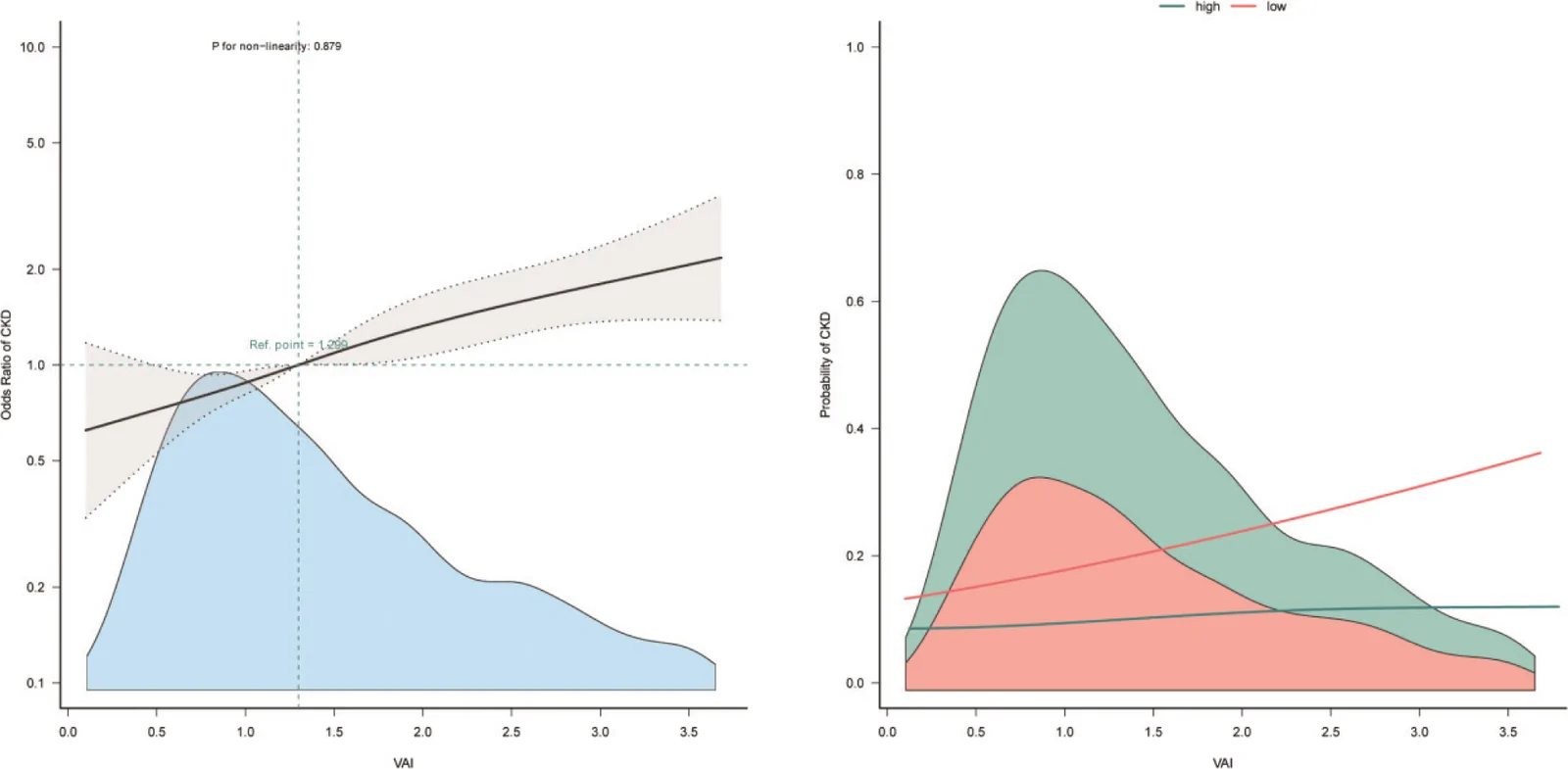
Restricted cubic spline analysis of the association between visceral adiposity index (VAI) and chronic kidney disease (CKD). (A) Dose–response relationship between VAI and CKD in the overall study population after adjustment for covariates included in Model 4. (B) Dose–response relationship between VAI and CKD stratified by dietary magnesium intake categories. The solid lines represent adjusted odds ratios (ORs), and the shaded areas indicate 95% confidence intervals (CIs). No evidence of non-linearity was observed in the overall population (*P* for non-linearity = 0.879).

The positive association between VAI and CKD was still observed in the low dietary magnesium intake group. Participants with high dietary magnesium intake on the other hand did not show an increase in CKD risk with increasing VAI, which further supports the potential modifying effect of dietary magnesium intake.

## Discussion

In this nationally representative study, we observed a significant relationship between the VAI and CKD among the U.S. adults. Importantly, this relation was significantly modified by dietary magnesium intake. Those with low dietary magnesium intake had higher odds of CKD associated with higher VAI, while this was not found for those with high dietary magnesium intake. Bamba et al. (37) showed that high VAI was associated with incident CKD in a large Chinese population, and Seong et al. (18) and Chen et al. (19) showed positive associations between VAI and incident CKD in males of Korean and Taiwanese populations, respectively. Our study extends these findings to a nationally representative and ethnically diverse U.S. population.

The association of VAI and CKD can be attributed to several biological mechanisms. It was reported that visceral adiposity leads to renal lipotoxicity (5), systemic inflammation (38, 39), oxidative stress (40, 41), endothelial dysfunction and activation of the renin–angiotensin–aldosterone system (42). These procedures can lead to “glomerular damage” and gradual deterioration of kidney function.

Our finding of a modifying effect of magnesium intake is biologically plausible. Magnesium is involved in many metabolic activities such as homeostasis of glucose, insulin signaling, antioxidant defense and activation of vitamin D (21). In the NHANES study (30), participants with the highest magnesium intake had the lowest insulin resistance and best metabolic profile. Due to the importance of insulin resistance and metabolic dysfunction in CKD development and progression, adequate magnesium intake may attenuate some of the adverse metabolic effects associated with visceral adiposity. Although the details of the mechanisms remain to be explored, our results indicate that dietary magnesium could affect the association between the visceral adiposity and kidney health. The results in this study must be used with caution, however, and should not be used as proof of a protective causal effect.

From a public health perspective, these findings highlight the potential importance of adequate dietary magnesium intake among individuals with elevated visceral adiposity. Consuming a diet rich in magnesium, such as a diet with more nuts, legumes, whole grains, and green leafy vegetables may represent a practical nutritional approach for populations with a higher risk of CKD. However, prospective cohort studies and randomized controlled trials are needed before the development of specific clinical recommendations (28–30).

## Strengths and limitations

Several limitations should be acknowledged. Firstly, a cross-sectional design makes it impossible to draw causal inferences from NHANES. Reverse causality is also a potential confounder, as CKD could affect dietary patterns, magnesium intake, body composition and metabolic status. However, in this study, the temporal sequence between exposure and outcome cannot be established. Secondly, dietary magnesium intake was measured by a single 24-h dietary recall interview. Although this approach is common in nutritional epidemiology, it may fail in obtaining usual dietary patterns for each participant and can contain recall bias and measurement errors. Thirdly, although we tried to adjust multiple demographic, lifestyle and clinical covariates, residual confounding cannot be completely ruled out. Data on medication use, some socioeconomic factors and renal-related comorbidities were not available in all NHANES survey cycles, and thus these factors were not fully addressed in the analyses.

Despite these limitations, this study has several important strengths. It utilized a large nationally representative sample of U.S. adults, incorporated complex survey weighting procedures, and employed multiple imputation to minimize bias related to missing data. To our knowledge, this is among the first studies to investigate the modifying role of dietary magnesium intake in the association between VAI and CKD in the U.S. population.

## Conclusion

In conclusion, our results show a significant positive association between VAI and CKD in a large sample of U.S. adults with low dietary magnesium intake. This association was not seen in the group of U.S. adults with high dietary magnesium intake. In addition, more randomized controlled studies or cohort studies are needed in the future to provide new evidence on the effect of dietary magnesium on VAI and CKD.

## Data Availability

All data generated or analyzed during this study are available from the corresponding author on reasonable request.
